# Deep Learning Network with Illuminant Augmentation for Diabetic Retinopathy Segmentation Using Comprehensive Anatomical Context Integration

**DOI:** 10.3390/diagnostics15212762

**Published:** 2025-10-31

**Authors:** Sakon Chankhachon, Supaporn Kansomkeat, Patama Bhurayanontachai, Sathit Intajag

**Affiliations:** 1 College of Digital Science, Prince of Songkla University, Songkhla 90110, Thailand; 6610035007@email.psu.ac.th; 2 Division of Computational Science, Faculty of Science, Prince of Songkla University, Songkhla 90110, Thailand; supaporn.k@psu.ac.th; 3 Department of Ophthalmology, Faculty of Medicine, Prince of Songkla University, Songkhla 90110, Thailand; patama103@yahoo.com.au

**Keywords:** diabetic retinopathy, semantic segmentation, anatomical context, domain generalization, data augmentation, transformer

## Abstract

**Background/Objectives:** Diabetic retinopathy (DR) segmentation faces critical challenges from domain shift and false positives caused by heterogeneous retinal backgrounds. Recent transformer-based studies have shown that existing approaches do not comprehensively integrate the anatomical context, particularly training datasets combining blood vessels with DR lesions. **Methods:** These limitations were addressed by deploying a DeepLabV3+ framework enhanced with more comprehensive anatomical contexts, rather than more complex architectures. The approach produced the first training dataset that systematically integrates DR lesions with complete retinal anatomical structures (optic disc, fovea, blood vessels, retinal boundaries) as contextual background classes. An innovative illumination-based data augmentation simulated diverse camera characteristics using color constancy principles. Two-stage training (cross-entropy and Tversky loss) managed class imbalance effectively. **Results:** An extensive evaluation of the IDRiD, DDR, and TJDR datasets demonstrated significant improvements. The model achieved competitive performances (AUC-PR: 0.7715, IoU: 0.6651, F1: 0.7930) compared with state-of-the-art methods, including transformer approaches, while showing promising generalization on some unseen datasets, though performance varied across different domains. False-positive returns were reduced through anatomical context awareness. **Conclusions:** The framework demonstrates that comprehensive anatomical context integration is more critical than architectural complexity for DR segmentation. By combining systematic anatomical annotation with effective data augmentation, conventional network performances can be improved while maintaining computational efficiency and clinical interpretability, establishing a new paradigm for medical image segmentation.

## 1. Introduction

Diabetic retinopathy (DR) represents the leading cause of preventable blindness among working-age populations in developed nations, affecting millions of diabetic patients worldwide [1]. The early and accurate detection of DR lesions, including microaneurysms (MAs), hemorrhages (HEs), hard exudates (EXs), and soft exudates (SEs), also known as cotton-wool spots (CWS), is crucial for timely intervention and preventing irreversible vision loss. However, visual diagnosis by ophthalmologists is inherently time-consuming, labor-intensive, and subject to inter-observer variability, creating an urgent need for automated diagnostic systems.

Current automated DR segmentation approaches can be categorized into traditional machine learning methods and deep learning (DL) techniques. Traditional approaches [2,3] rely on handcrafted features, thresholding, and mathematical morphology but suffer from poor generalization and require extensive manual parameter tuning. DL-based methods, particularly Convolutional Neural Networks (CNNs) [4,5,6,7,8], have shown remarkable success but face three critical limitations that severely limit their clinical deployment.

**First, the domain shift problem:** Models trained on one dataset exhibit significant performance degradation when applied to images from different cameras, institutions, or patient populations [9,10,11]. Variations in imaging protocols, camera specifications, and photometric characteristics across clinical settings all contribute to this problem.

**Second, background-induced false positives:** Retinal images contain complex anatomical structures that can be mistaken for pathological lesions, leading to diagnostic errors. For instance, the optic disc (OD) often presents similar color characteristics to EXs, while blood vessels may be confused with HEs or MAs.

**Third, incomplete anatomical context utilization:** Huang et al. noted in their seminal RTNet work [5] “as no fundus dataset is annotated with both vessels and DR lesions, the DL-based lesion segmentation task no longer extracts vessel features separately.” They also concluded that “pathological connections have not received enough attention” and that their work represented “the first trial that utilizes vascular information for deep based fundus lesion segmentation.” However, their approach was limited to vessel information alone, without comprehensive retinal anatomical context.

This paper addresses these fundamental limitations through a paradigm shift from architectural complexity to comprehensive data enhancement. Rather than developing sophisticated transformer networks, this work demonstrates that conventional architectures achieve competitive performance when provided with appropriate anatomical context and effective domain adaptation strategies.

**The core innovation lies in systematic anatomical context integration:** Extending beyond the vessel-only approach pursued in [5], this study develops the first comprehensive training framework that systematically combines DR lesions with complete retinal anatomical structures, including OD, fovea (FV), blood vessels, and retinal boundaries as explicit contextual background classes.

**The secondary innovation addresses domain shift through realistic data augmentation:** An illumination-based augmentation method simulates diverse camera characteristics using color constancy principles, exposing models to realistic photometric variations during training without requiring target domain data. Comprehensive experimental validation demonstrates the effectiveness of the proposed approach across multiple dimensions:

- **Cross-dataset generalization:** Training on the Indian Diabetic Retinopathy Image Dataset (IDRiD) [12] and evaluation on the unseen test datasets of Diabetic Retinopathy Grading and Segmentation Challenge (DDR) [13] and Tianchi Diabetic Retinopathy (TJDR) [14] validate robustness across different camera systems and patient populations.
- **Ablation studies:** Systematic analysis isolates the contributions of anatomical context integration and illumination augmentation.
- **Comparative analysis:** Performance comparison against state-of-the-art methods including recent transformer approaches demonstrates superior accuracy and efficiency.

The objectives of this study are threefold: first, to develop a comprehensive training framework that systematically integrates complete retinal anatomical structures (optic disc, fovea, blood vessels, and retinal boundaries) with DR lesion annotations, addressing the gap in existing approaches that lack comprehensive anatomical context modeling; second, to design and validate an illumination-based data augmentation strategy grounded in color constancy principles that simulates realistic photometric variations across diverse fundus camera systems, thereby improving model robustness against domain shift without requiring target domain data; third, to demonstrate through rigorous experimental validation across multiple datasets that data enrichment through anatomical context integration and effective augmentation can achieve competitive performance with established network architectures, providing a clinically interpretable and computationally efficient alternative to increasingly complex architectural designs. By realizing these objectives, we aim to establish a new paradigm for DR segmentation that prioritizes comprehensive data strategy over architectural complexity while maintaining clinical interpretability and ease of deployment.

Qualitatively, the framework offers several clinical advantages: (1) reduced false positives through anatomical context awareness, (2) improved interpretability compared to attention-based methods, and (3) a systematic approach to dataset enhancement that can benefit other medical imaging tasks.

The main contributions of this work include the following:

- **Systematic anatomical context integration:** Development of the first comprehensive training dataset combining DR lesions with complete retinal anatomy, extending beyond existing vessel-only approaches to include comprehensive background structures.
- **Paradigm shift demonstration:** Empirical evidence that comprehensive data enhancement outperforms architectural complexity, challenging the trend toward increasingly complex models in medical imaging.
- **Domain adaptation innovation:** Novel illumination-based augmentation that effectively simulates real-world photometric variations without requiring target domain annotations.
- **Clinical validation:** Extensive cross-dataset evaluation demonstrating practical applicability across diverse clinical settings with strong generalization compared to existing methods.

## 2. Related Works and Literature Review

The literature review is organized thematically to highlight the evolution of techniques and identify persistent gaps that motivated the proposed approach.

### 2.1. Traditional Approaches and Early Deep Learning Methods

Early DR lesion segmentation methods relied on traditional computer vision techniques, including thresholding, mathematical morphology, and handcrafted feature extraction [2,3]. While these approaches provided interpretable results, they suffered from poor generalization across different datasets and required extensive manual parameter tuning by domain experts. The computational complexity and limited adaptability of these methods made them unsuitable for large-scale clinical deployment.

The emergence of DL revolutionized medical image analysis, with CNNs demonstrating superior performance in various segmentation tasks. Khojasteh et al. [15] pioneered the use of four-layered CNNs to detect exudates, microaneurysms, and hemorrhages, while Eftekhari et al. [16] proposed a two-stage CNN approach specifically for microaneurysm detection. However, these early DL approaches treated DR lesion segmentation as an isolated task without considering the broader anatomical context of retinal images.

### 2.2. Foundation Architectures and Their Evolution

**U-Net and Encoder–Decoder Architectures:** Ronneberger et al. [17] introduced U-Net, establishing the encoder–decoder architecture with skip connections as the foundation for medical image segmentation. This architecture enabled precise localization while maintaining contextual information, becoming the de facto standard for medical imaging applications due to its effectiveness with limited training data.

**Advanced Encoder–Decoder Variants:** Building upon the success of U-Net, Zhou et al. [18] developed UNet++, including nested and dense skip pathways to bridge semantic gaps between encoder and decoder features. Chen et al. [19] contributed DeepLabV3+ with atrous separable convolution, combining spatial pyramid pooling with an encoder–decoder structures for multi-scale context aggregation. Gu et al. [20] proposed CE-Net, adding context encoder modules for enhanced semantic understanding.

**Multi-Scale and Attention Mechanisms:** Zhao et al. [21] introduced PSPNet with pyramid pooling modules for global context aggregation. Recent works have incorporated attention mechanisms. Bo et al. [22] developed scale-aware attention blocks for adaptive lesion detection across different scales.

### 2.3. Transformer-Based and Advanced Architectures

**Relation Modeling:** Huang et al. [5] developed RTNet (Relation Transformer Network), the first model to leverage transformer architecture for capturing long-range dependencies between different DR lesion types. Their work identified a critical gap: “as no fundus dataset is annotated with both vessels and DR lesions, the DL-based lesion segmentation task no longer extracts vessel features separately.” They noted that “pathological connections have not received enough attention” and presented “the first trial that utilizes vascular information for deep based fundus lesion segmentation.”

**Prior-Guided Attention:** Xu et al. [23] proposed prior-guided attention fusion transformers that incorporate anatomical priors to guide attention mechanisms. While this approach showed promise, it relied primarily on complex attention computations rather than systematic data enhancement.

**Specialized Architectures:** Pavani et al. [24] introduced RILBP-YNet, combining rotation invariant local binary patterns with Y-shaped architecture for multiclass lesion segmentation. These approaches demonstrated progression toward a more sophisticated architecture at the cost of computational complexity and interpretability.

### 2.4. Domain Adaptation and Generalization Approaches

**Domain Shift Challenges:** Despite architectural advances, domain shift remains a persistent challenge. Lyu et al. [9] proposed AADG (Automatic Augmentation for Domain Generalization) for retinal image segmentation, while Che et al. [10] and Galappaththige et al. [11] explored domain generalization for DR classification. However, these approaches primarily focused on geometric and intensity transformations without addressing the fundamental lack of anatomical context in training data.

**Data Augmentation Strategies:** Traditional augmentation techniques include geometric transformations (rotation, flipping, scaling) and intensity modifications (brightness, contrast adjustments). While effective for basic generalization, these methods do not address the photometric variations introduced by different fundus camera systems and imaging protocols.

Recent advances in generative AI have explored diffusion models for medical image augmentation. Lakas et al. [25] demonstrated that diffusion models can enhance DR grading by generating synthetic training samples. However, these approaches were developed for classification tasks where image-level labels suffice. For pixel-precise segmentation, diffusion-based augmentation presents significant challenges. The generative process may destroy the fine details of small lesions such as microaneurysms, and synthesized images often lack the anatomical fidelity required for accurate boundary delineation. These limitations motivate our illumination-based approach, which preserves original structures while simulating realistic photometric variations.

### 2.5. Federated Learning for Diabetic Retinopathy

The growing concerns surrounding medical data privacy and the fragmentation of healthcare datasets have catalyzed the adoption of federated learning (FL) in DR research. Mao et al. [26] proposed a comprehensive FL framework that jointly addresses DR grading and multi-lesion segmentation without requiring centralized data aggregation. Their adaptive α-Fed algorithm comprises two main components, a local client model and a client-specific global model. It adaptively tunes the aggregate weighted α from round to round based on performance, balancing site-specific knowledge with information from other datasets. It marks a significant advancement in cross-domain federated optimisation, achieving competitive performances across heterogeneous datasets while preserving patient confidentiality and institutional data sovereignty.

This privacy-preserving paradigm aligns with earlier efforts, such as those of Sheller et al. [27], who demonstrated the feasibility of FL for brain tumor segmentation across multiple institutions, and Li et al. [28], who explored personalized FL strategies for medical image classification. Additionally, Nielsen et al. [29] proposed a homomorphic encryption-enhanced FL system for DR classification, significantly reducing computation and data transfer while protecting against gradient inversion attacks. These studies collectively underscore the potential of FL to overcome data silos and enable collaborative learning in sensitive clinical domains.

Nevertheless, our findings suggest that well-designed centralized data augmentation techniques can yield comparable performance to federated learning approaches for specific lesion types, with particular advantages in detecting small, challenging lesions such as microaneurysms and hemorrhages when domain-specific priors are incorporated. This highlights the importance of balancing privacy-preserving strategies with methodological flexibility in DR analysis.

### 2.6. Critical Gaps in Current Approaches

**Inadequate Integration of Anatomical Context:** Notwithstanding the acknowledgment by Huang et al. [5] regarding the significance of vascular information, there exists a deficiency in the systematic integration of comprehensive retinal anatomical structures (including the OD, FV, blood vessels, and retinal boundaries) with DR lesions within a cohesive training framework. Subsequently, Xu et al. [23] introduced a notable advancement by employing pre-trained vascular masks as prior knowledge to facilitate the segmentation of lesions.

**Background Structure Ambiguity:** Current methods lack explicit mechanisms to differentiate between pathological lesions and normal anatomical structures. The resemblances between ODs and exudates, and blood vessels and HEs, continue to cause false-positive segmentations.

**Domain Shift Vulnerability:** Existing augmentation strategies do not adequately simulate the realistic photometric variations encountered across different clinical settings, limiting model generalization to unseen domains.

**Architectural Complexity vs. Performance Trade-Offs:** Recent transformer-based approaches have improved performances but at significant computational cost, potentially limiting their clinical deployment. The positive relationship between architectural complexity and performance gains remains unclear.

### 2.7. Research Motivation and Positioning

The comprehensive literature review revealed a clear research gap: while recent works have explored sophisticated architectures and attention mechanisms, the fundamental challenge of inadequate training data remains unaddressed. Huang et al. [5] observed that the lack of datasets combining vessels with DR lesions highlighted a broader issue—the absence of comprehensive anatomical context in existing training frameworks.

This paper addresses these limitations through a systematic approach that prioritizes data enhancement over architectural complexity. By developing comprehensive anatomical context integration and realistic domain adaptation strategies, this work demonstrates that conventional architectures can produce competitive performances when provided with appropriate training data and augmentation strategies.

**Clear Separation Between Existing Work and Novel Contributions:** The literature review established that while existing approaches have explored various architectural innovations and limited anatomical integration, no prior work has systematically combined comprehensive retinal anatomical structures with DR lesions in a unified training framework. The proposed illumination-based augmentation represents a novel approach to domain adaptation that goes beyond traditional geometric and intensity transformations to simulate realistic photometric variations across different imaging systems.

## 3. Materials and Methods

This section details the proposed DL framework for multi-lesion DR segmentation. It begins by outlining the datasets utilized in this study and their preparation, followed by an emphasis on two key strategies: the generation of refined background subclass annotations to address heterogeneous backgrounds and reduce false positives, and an innovative illumination-based data augmentation technique to enhance model generalization against domain shift.

### 3.1. Datasets

This study leverages a combination of publicly available retinal fundus image datasets for both DR lesion segmentation and innovative illumination-based data augmentation. A detailed description of each dataset and its specific role in our research is provided below.

#### 3.1.1. Segmentation and Generalization Assessment Datasets

For the primary task of DR lesion segmentation and comprehensive generalization assessment, three widely recognized public datasets were utilized: the IDRiD [12], DDR [13] and TJDR [14] datasets.

- **IDRiD:** The dataset designated for segmentation encompasses 81 high-resolution retinal images, which have been meticulously annotated at the pixel level for the OD and four distinct DR lesions: MAs, HEs, EXs, and CWSs. In light of its superior pixel-level annotations, the IDRiD is regarded as the benchmark for the training and evaluation of our proposed segmentation methodology. For the purposes of experimentation, this dataset is traditionally partitioned into 54 images allocated for training and 27 images reserved for testing.
- **DDR Dataset:** The full DDR dataset comprises 13,673 retinal images, primarily designed for grading DR severity, ranging from No DR to Proliferative DR. For segmentation tasks within this study, a subset of 757 images is utilized, providing segmentation annotations for four lesion types: MA, HE, EX, and CWS. This segmentation subset is partitioned into 383 images for training, 225 images for testing, and 149 images for validation. This dataset is employed as an unseen test set specifically to assess the generalization capability of our proposed method. Its large size and distinct image characteristics provide a robust test for unseen data distributions.
- **TJDR Dataset:** The TJDR dataset consists of a collection of 561 retinal fundus images, specifically curated for DR analysis. A notable characteristic of this dataset is that its images were captured using a Zeiss CLARUS 500 and Topcon cameras, which provides a significantly different Field of View (FoV) compared to the camera used for the IDRiD dataset. This distinct FoV contributes significantly to the domain shift challenge, making TJDR an additional unseen test set to further validate the robustness and generalization performance of our model across diverse clinical image acquisition protocols. The TJDR dataset is divided into 448 images for training and 113 images for testing.

#### 3.1.2. Illuminant Augmentation Reference Datasets

To facilitate the proposed illumination-based data augmentation, four additional public datasets were employed as sources of diverse illuminant appearances: STARE [30], Chase_DB1 [31], HRF [32], and DRIVE [33]. These datasets are predominantly focused on vessel segmentation and exhibit varied imaging characteristics, making them ideal for simulating real-world photometric variations.

- **STARE Dataset:** This dataset contains 20 retinal images, equally split between normal (10 images) and pathological cases (10 images).
- **Chase_DB1 Dataset:** Collected from the Child Heart and Health Study in Beijing, this dataset provides 28 retinal images, with vessel ground truth annotations obtained by two independent observers.
- **HRF Dataset (High-Resolution Fundus Image Database):** Comprising 45 images, HRF is categorized into three groups: healthy, DR, and glaucomatous retinopathy.
- **DRIVE Dataset (Digital Retinal Images for Vessel Extraction):** This dataset includes 40 retinal images, with 7 abnormal and 33 normal cases, and is widely used for vessel segmentation research.

Collectively, all 113 images from these four datasets were utilized to extract diverse illuminant appearances. This extensive collection serves as the reference pool for our novel illumination augmentation method, ensuring that our training data was exposed to a wide range of realistic photometric variations.

#### 3.1.3. Dataset Utilization Summary

In conclusion, owing to its meticulous pixel-level annotations of lesions, the IDRiD dataset is utilized as the foundational dataset for the training and evaluation of our proposed segmentation framework. The DDR and TJDR datasets are judiciously utilized as unseen test sets to rigorously assess the generalization ability and robustness of the model on large-scale, independently sourced datasets characterized by a diverse array of features. The STARE, Chase_DB1, HRF, and DRIVE datasets are collectively employed to provide vessel resources and serve as reference materials for the generation of varied illuminant appearances, thereby substantially enhancing the IDRiD training dataset and improving the resilience of the model against domain shifts. Table 1 provides a comprehensive summary of all datasets utilized in this study, including their sources, characteristics, and specific roles in our experimental framework.

### 3.2. Data Preparation and Annotated Background Subclasses

To address the challenge of heterogeneous retinal image backgrounds and improve the precision of DR lesion segmentation, we developed a comprehensive approach to create detailed background subclass annotations. This process involved leveraging existing datasets and employing a two-stage annotation strategy.

**Figure 1:**
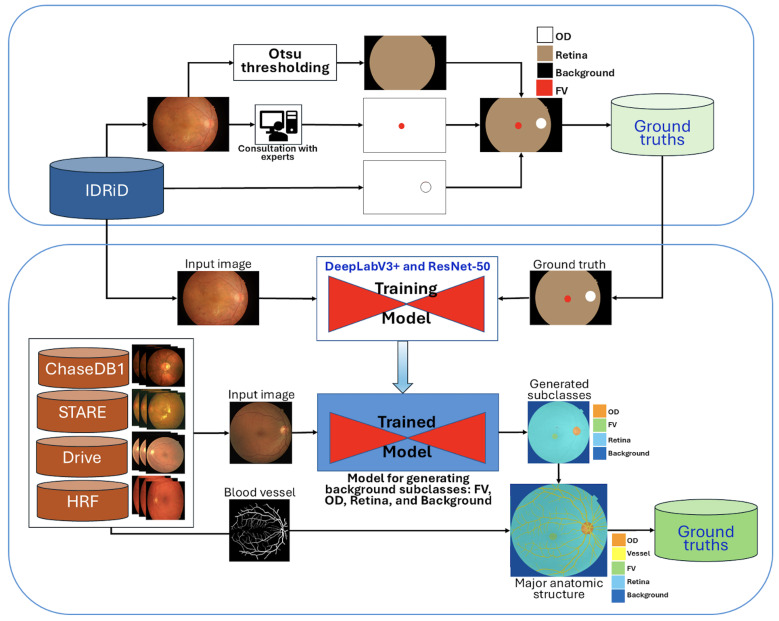
A conceptual schematic delineating the **process of constructing the ground truth**, which illustrates input images alongside associated background subclasses (FV, OD, and retina) as produced ground truth in the upper section. The lower section shows how these generated subclasses are combined with blood vessel segmentations to delineate the principal anatomical structures: OD, blood vessel, FV, retina, and background.

Initially, the IDRiD dataset was utilized, providing pixel-level annotations for the OD and four DR lesion types. To generate the retinal region, Otsu thresholding was applied as described in [34]. Subsequently, the retinal and OD annotations were combined, and the FV region was manually edited using MATLAB’s Image Labeler [35] (MATLAB software, https://www.mathworks.com/products/matlab.html (accessed on 27 October 2025)), with careful investigation and validation by experienced ophthalmologists. The resulting background subclass annotations, including OD, FV, and the general retinal area, are conceptualized in Figure 1.

A pre-trained DeepLabV3+ model with a ResNet-50 backbone, as depicted in Figure 1, was then trained using the 81 segmentation images from the IDRiD dataset. This trained model was subsequently employed to generate initial background subclass annotations (FV, OD, retina, and background) for the public datasets STARE, Chase_DB1, HRF, and DRIVE.

To further enhance the anatomical completeness, the subclasses generated from these four datasets were overlaid with their corresponding blood vessel segmentations. The lower section in Figure 1 illustrates this comprehensive production process, where generated subclasses are combined with blood vessel information to form a more complete set of major anatomic structures. All newly generated background subclass annotations were meticulously investigated and manually edited using the Image Labeler App to correct any inaccuracies.

**Figure 2:**
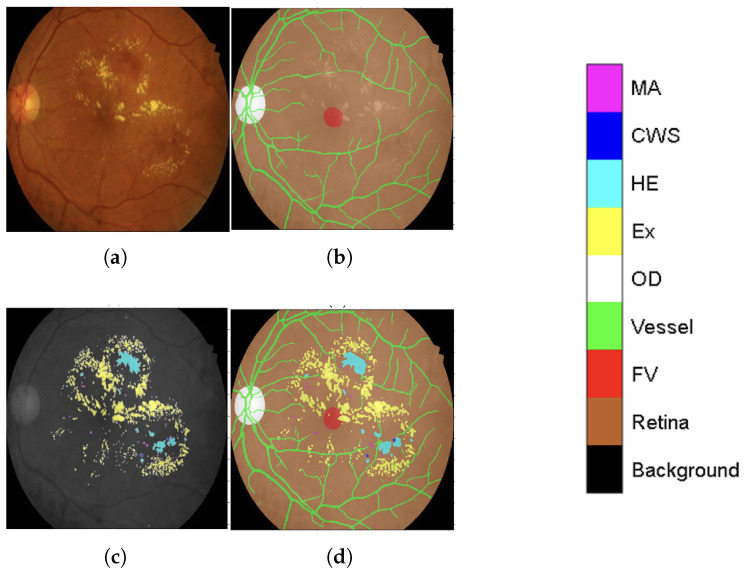
Generating contextual labeling to enhance DR segmentation: (**a**) Input image; (**b**) Major anatomic structures from the generated model; (**c**) Four lesion annotations from IDRiD: MA, CWS, HE, and EX; (**d**) Combining the anatomic structure with the four lesions.

Finally, these four public datasets (STARE, Chase_DB1, HRF, and DRIVE) with their newly established major anatomic structure annotations were used to train a dedicated “major anatomic structure model”. This model utilized the same DeepLabV3+ architecture with a ResNet-50 backbone (shown conceptually in Figure 1), but with its output block modified to predict five classes (OD, FV, vessel, retina, and background). Upon achieving the target validation performance, this specialized model was then used to generate a consistent set of major anatomic structure annotations for the entire IDRiD dataset, as exemplified in Figure 2b.

This meticulously annotated IDRiD dataset, now enriched with contextual back-ground labeling, significantly improves segmentation capabilities by providing a clearer distinction between lesions (original annotation as seen in Figure 2c) and heterogeneous background structures. The improved IDRiD dataset (Figure 2d) was then divided into training and validation sets following the same separation as the original dataset, resulting in 54 improved training data samples used to train the final network model by adjusting its output block to nine classes (four DR lesions + five background subclasses).

#### Main Steps for Anatomical Context Integration

To clarify our approach, the comprehensive anatomical context integration process illustrated in Figure 1 and Figure 2 can be summarized in four main sequential steps:

- **Step 1:** Initial Background Subclass Annotation. We began with the IDRiD dataset, which provided pixel-level annotations for the optic disc and four DR lesion types. We generated additional anatomical annotations, including the retinal region (using Otsu thresholding) and fovea (through manual editing validated by experienced ophthalmologists). These initial annotations established the foundation for the full anatomical modeling.
- **Step 2:** Major Anatomical Structure Model Development. A DeepLabV3+ model with ResNet-50 backbone was trained on the IDRiD dataset to predict four classes: optic disc, fovea, retina, and background. This model was then applied to four public vessel datasets (STARE, Chase_DB1, HRF, DRIVE) to generate initial anatomical structure annotations. The generated annotations were overlaid with existing vessel segmentations and manually refined using MATLAB’s Image Labeler tool to ensure accuracy. This refinement process involved review by two trained graders, achieving an inter-observer agreement of an F1-score = 0.9754. The final validation was carried out by an ophthalmologist with more than 10 years of experience.
- **Step 3:** Comprehensive Anatomical Annotation for IDRiD. The major anatomical structure model, trained on the four vessel datasets enriched with anatomical annotations, was then applied back to the entire IDRiD dataset to generate consistent, comprehensive anatomical structure masks. This reverse application ensured that the IDRiD training data contained complete anatomical context information derived from diverse imaging sources.
- **Step 4:** Multi-Class Integration for Lesion Segmentation. Finally, the anatomical structure annotations (optic disc, fovea, vessels, retina, background) were combined with the original DR lesion annotations (microaneurysms, hemorrhages, hard exudates, cotton-wool spots) to create a unified nine-class training dataset. This integration enabled the model to simultaneously learn lesion patterns and anatomical context, reducing false positives by explicitly modeling structures that could be confused with pathological features.

Through this systematic process, our framework leverages anatomical context to improve lesion discrimination. The explicit modeling of background structures as separate classes enables the network to learn distinctive features for each anatomical region, thereby reducing the misclassification of normal structures as pathological lesions.

### 3.3. Illuminant Augmentation

Given the limited size of available medical imaging datasets and the inherent variability in retinal image distributions obtained from different acquisition systems, data augmentation is a crucial technique that can expand training data synthetically and prevent overfitting. Common augmentation strategies encompass both spatial transformations (e.g., random rotations up to ±15 degrees, flipping) and tonal transformations, which alter brightness, contrast, and color balance. Tonal variations [36] are particularly prevalent in retinal images, often the results of diverse imaging devices, lighting conditions, and patient characteristics. Studies have consistently shown that images of the same retina can appear distinctly different when captured by different fundus cameras [37,38], with inconsistencies attributed to device-specific color rendering and photo-graphic conditions. While such tonal enhancements can aid human clinical interpretation, they can detrimentally affect the performances of Deep Neural Networks (DNNs) if not properly addressed. Therefore, to improve the generalization capabilities of DNNs across diverse imaging conditions, it is essential to incorporate comprehensive tonal augmentation by exposing DNNs to a wide range of real-world variations during training.

**Figure 3:**
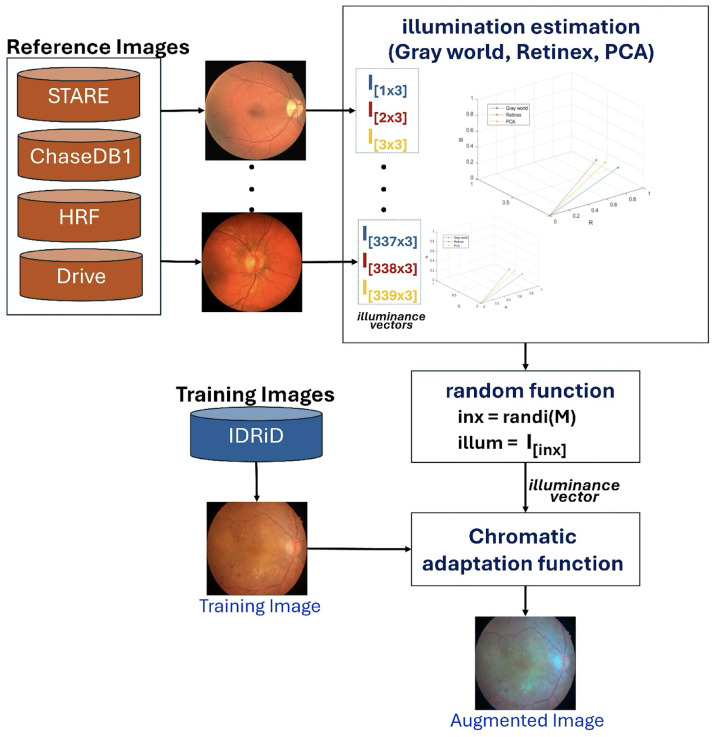
The diagram shows the illuminant augmentation process used to augment 54 training IDRiD images by providing reference images from four datasets: STARE, Chase_DB1, HRF, and DRIVE.

Our proposed scheme introduces an innovative illumination-based data augmentation method designed to transfer the appearance of colors in reference images to the training data. As detailed in Table 2, the 54 IDRiD training images are augmented by mimicking image acquisition from other datasets (STARE, Chase_DB1, HRF, and DRIVE). This transformation process, depicted in Figure 3, leverages principles of color constancy [39] to reproduce training images with varied illuminance properties.

**Figure 4:**
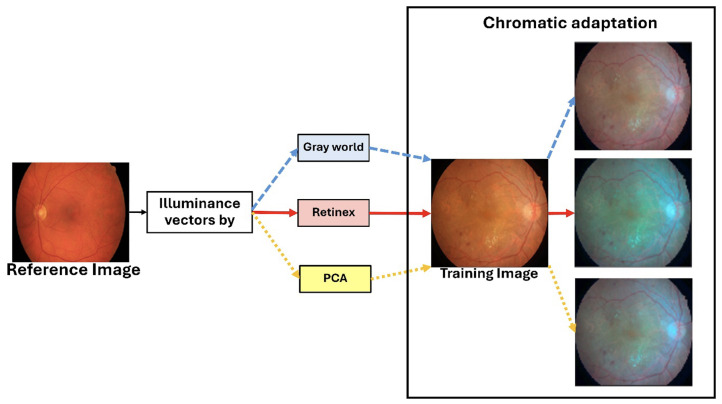
The enhanced illumination is derived from the reference HRF image through the application of Gray World, Retinex, and PCA methodologies to adjust the IDRiD image, which yields the augmented images presented in the final column.

The augmentation process begins by pre-calculating an illuminance matrix ($I_{\left[M\times 3\right]}$). This matrix is derived from 113 reference images collected from the STARE, Chase_DB1, HRF, and DRIVE datasets. For each of these 113 images, illuminance vectors are estimated using three established color constancy algorithms: Gray World, Retinex, and Principal Component Analysis (PCA) [40], resulting in a total of 339 illuminance vectors (M=113×3). During training, a distinct illuminance vector is uniformly and randomly selected from this pre-calculated matrix ($I_{\left[M\times 3\right]}$) for each of the 54 IDRiD training images. The selected training image is then rendered using a chromatic adaptation function, which applies the chosen illuminance vector to generate an augmented image with a different visual appearance. As illustrated in Figure 4, this process effectively simulates the scenario where an IDRiD image is captured by a different camera, such as one used for HRF images.

#### Rationale for Illumination-Based Augmentation

The fundamental motivation for investigating illumination-based augmentation stems from the observation of a critical weakness in clinical retinal imaging—fundus images acquired from different cameras, institutions, and imaging protocols exhibit substantial variations in photometric characteristics, including illumination intensity, color temperature, and spectral response. These variations represent a primary source of domain shift, causing models trained on one dataset to exhibit significant performance degradation when applied to images from different acquisition systems. Traditional color-based augmentation techniques, such as random brightness and contrast adjustments, generate arbitrary transformations that may not reflect realistic imaging variations, and may potentially introduce non-physiological color distributions that could mislead the model.

Our illumination-based augmentation strategy addresses this limitation by synthesizing realistic photometric variations grounded in the principles of color constancy theory. Illumination characteristics are extracted from multiple established vessel segmentation datasets (STARE, DRIVE, HRF, Chase_DB1). The strategy creates a reference pool of realistic illumination profiles acquired by different camera systems in diverse imaging conditions. During training, these profiles are applied to the IDRiD images through chromatic adaptation, effectively simulating scenarios where each training image was captured by different camera systems. This approach exposes the model to a broader spectrum of realistic imaging conditions without requiring actual images from multiple sources, thereby encouraging the learning of illumination-invariant features that are more robust to domain shift. The use of established color constancy algorithms (Gray World, Retinex, and PCA) ensures that the augmented images maintain a physiologically plausible color distributions while substantially increasing photometric diversity.

### 3.4. Segmentation Model Architecture and Training

Our semantic segmentation framework utilizes DeepLabV3+ [19] with a ResNet-50 [41] backbone, as illustrated conceptually in Figure 1. All input images were resized to 1024 × 1024 pixels for consistent processing. The final classification block of the network was adapted based on the specific segmentation task:

- Model-1: Predicts 4 classes (OD, FV, retina, and background).
- Model-2 (Major Anatomic Structure Model): Predicts five classes (for OD, FV, vessel, retina, and general background), augmented with 81 IDRiD images during training.
- Model-3: Predicts nine classes (four DR lesions + four background subclasses + general background), augmented with 113 combined images from STARE, Chase_DB1, HRF, and DRIVE datasets.

Training was conducted in a two-stage process to optimize segmentation and handle class imbalance effectively:

- **Stage 1 (Cross-Entropy Loss):** Initially, models were trained using the standard cross-entropy loss function [42], which is defined as follows:
  (1) $$loss=\frac{1}{N}\sum_{n=1}^{N}\sum_{i=1}^{C}w_{i}\;t_{ni}\ln y_{ni}$$
  where *N* is the number of images, *C* is the number of classes, $w_{i}$ is the weight for class *i*, $t_{ni}$ represents whether the *n*-th image pixel belongs to the *i*-th class (ground truth), and $y_{ni}$ is the output probability (from softmax) for image *n* belonging to class *i*. This stage helps the model to initially capture all relevant DR lesions.
- **Stage 2 (Generalized Tversky Loss):** In the second stage, the cross-entropy loss was replaced with the generalized Tversky loss function [43], which is based on the Tversky index. The Tversky index ($TI_{c}$) for measuring overlap between a predicted segmentation *Y* and corresponding ground truth *T* for class *c* is given by
  (2) $$TI_{c}=\frac{\sum_{m=1}^{M}Y_{cm}T_{cm}}{\sum_{m=1}^{M}Y_{cm}T_{cm}+\alpha \sum_{m=1}^{M}Y_{cm}T_{\bar{c}m}+\beta \sum_{m=1}^{M}Y_{\bar{c}m}T_{cm}}$$
  where *c* corresponds to the target class, $\bar{c}$ corresponds to not being in class *c*, *M* is the number of elements (pixels), and α and β are weighting factors that control the contribution of false positives and false negatives, respectively. The overall loss *L* across *C* classes is defined as follows:
  (3) $$L=\sum_{c=1}^{C}\left(1-TI_{c}\right)$$

This two-stage approach leveraging Tversky loss is particularly beneficial for addressing class imbalance, a common issue in medical image segmentation where lesion pixels are typically far fewer than background pixels.

The training regimen employing the cross-entropy loss function for each model exhibited variability in the number of epochs: specifically, 300, 400, and 600 epochs were allocated for Model-1, Model-2, and Model-3, respectively. In contrast, in order to ascertain the optimal performance utilizing the Tversky loss, each model underwent a training duration of 100 epochs. A consistent learning rate of 0.001 was applied across all models, with parameters updated using Stochastic Gradient Descent with Momentum (SGDM) and a momentum value of 0.90. All models were trained on a GeForce RTX™ 3090 Ti GPU with 24 GB of memory, using a batch size of 6.

### 3.5. Evaluation Metrics

To comprehensively assess the performances of our semantic segmentation models, which operate on retinal images where pathological regions typically occupy less than 5% of the total area [44], a robust evaluation framework was essential. This class imbalance necessitates the use of a complementary set of metrics that can accurately reflect the performance of a model beyond simple pixel accuracy. This section presents our comprehensive evaluation framework, which utilizes seven widely recognized metrics: Accuracy, Intersection over Union (IoU), Precision, Recall, Area Under the ROC Curve (AUC-ROC), Area Under the Precision–Recall Curve (AUC-PR), and F1-Score.

- **Accuracy:** Measures the overall pixel classification correctness. Due to class imbalance, this metric can be misleadingly high if the model correctly classifies a large number of background pixels but fails to identify lesions. Accuracy was determined as follows:
  (4) $$accuracy=\frac{TP+TN}{TP+TN+FP+FN}$$
  In the above formula:
  - **TP (True Positive):** Number of pixels correctly identified as part of a target class (overlapping with ground truth).
  - **TN (True Negative):** Number of pixels correctly identified as belonging to the non-interest (background) class.
  - **FP (False Positive):** Number of pixels incorrectly identified as part of a target class.
  - **FN (False Negative):** Number of pixels that are part of a target class but incorrectly identified as non-interest.
- **Intersection over Union (IoU):** Also known as the Jaccard index, IoU measures the spatial overlap between the predicted segmentation mask and the ground truth mask. It provides a direct geometric interpretation of segmentation quality and is a standard metric for benchmarking segmentation models, as it is unaffected by the abundance of true negative pixels [45].
  (5) $$IoU=\frac{TP}{TP+FP+FN}$$
- **Precision:** Quantifies the proportion of predicted lesion pixels that are actually correct. A high Precision score is critical in DR segmentation as it minimizes false positives, reducing unnecessary alarms or follow-up procedures for patients [46].
  (6) $$Precision=\frac{TP}{TP+FP}$$
- **Recall:** Also known as sensitivity, Recall measures the proportion of actual lesion pixels that are correctly identified. A high Recall score is vital for a robust screening system, as it minimizes false negatives, ensuring that no patient with DR is undiagnosed [47].
  (7) $$Recall=\frac{TP}{TP+FN}$$
- **F1-Score:** The F1-score is a metric that evaluates the balance between Precision and Recall in segmented objects. It is particularly important in segmentation tasks where overall accuracy is more critical than the precision of boundaries.
  (8) $$F1=\frac{2\cdot Precision\cdot Recall}{Precision+Recall}$$
  A high F1-score indicates that the model performs well in identifying relevant regions while minimizing false positives and false negatives.
- **Area Under the ROC Curve (AUC-ROC):** AUC-ROC evaluates the ability of a model to distinguish between the positive and negative classes across all possible thresholds.
  (9) $$AUC-ROC=\sum_{i=1}^{n}\frac{\left(FPR_{i}-FPR_{i-1}\right)\left(TPR_{i}+TPR_{i-1}\right)}{2}$$
  where:
  - *n* = number of threshold points;
  - $TPR_{i}$ stands for a true positive rate, $TPR_{i}=\frac{TP_{i}}{TP_{i}+FN_{i}}$ equal to sensitivity at threshold *i*;
  - $FPR_{i}$ stands for a false positive rate, $FPR_{i}=\frac{FP_{i}}{FP_{i}+TN_{i}}=1$—Specificity at threshold *i*;
  - Points ordered by increasing FPR (from (0,0) to (1,1)).
  AUC-ROC could be misleadingly optimistic for DR-lesion segmentation due to background pixel dominance. On 97% background pixels, a random classifier achieves an AUC-ROC ≈0.5, but a slightly better than random classifier can return an AUC-ROC >0.9 while having poor lesion detection (IoU <0.2) [48,49].
- **Area Under Precision–Recall Curve (AUC-PR):** This metric provides a holistic view of the trade-off between Precision and Recall at various classification thresholds.
  (10) $$AUC-PR=\sum_{i=1}^{n}\frac{\left(r_{i}-r_{i-1}\right)\left(P_{i}-P_{i-1}\right)}{2}$$
  where:
  - $r_{i}$ denotes Recall at threshold *i* (sorted in ascending order);
  - $P_{i}$ equals Precision at threshold *i*.

For highly imbalanced datasets like ours, the AUC-PR is a more informative and reliable indicator of model performance than the AUC-ROC, as it focuses specifically on the ability of the model to correctly identify the positive class (lesions) without being influenced by a large number of true negatives [50,51]. For DR lesions with <5% prevalence, the AUC-PR provides better discrimination between model performances [52].

DR-lesion segmentation evaluation requires a multi-metric approach due to the inherent severe class imbalance. IoU serves as the primary metric for spatial accuracy, complemented by Recall for clinical sensitivity and the AUC-PR for threshold-independent assessment. Traditional metrics like Accuracy and the AUC-ROC provide limited value and can be misleading. The proposed framework effectively reconciles clinical demands with technical efficacy, which is essential for the precise delineation of intricate anatomical features and DR lesions in empirical findings.

All comparative analyses employ these established metrics to evaluate model performance. Statistical significance testing with *p*-values and bootstrap confidence intervals is conducted where applicable (see Section 4.2), providing rigorous quantification of performance differences across methods.

## 4. Experimentation Results

This section presents the results of our experiments, providing a comprehensive analysis of the proposed framework’s performance. We first detail the results of the baseline models and then evaluate the impact of our two primary contributions: the incorporation of annotated background subclasses and the illumination-based data augmentation. The performance of the final proposed model is then compared against state-of-the-art methods across multiple datasets to demonstrate its strong generalization capabilities.

### 4.1. Ablation Studies

To systematically evaluate the individual and combined contributions of the proposed innovations, comprehensive ablation studies were conducted using rigorous experimental protocols. The ablation analysis was designed to isolate and quantify the specific impact of two key components—(1) comprehensive anatomical context integration and (2) illumination-based domain adaptation—addressing the fundamental research questions posed in this work.

All experiments employed a standardized DeepLabV3+ architecture with ResNet-50 backbone to ensure fair comparison and eliminate architectural bias. The consistent use of the two-stage training protocol (cross-entropy followed by Tversky loss optimization) across all model variants ensured that performance differences could be attributed solely to the proposed innovations rather than training methodology variations. The ablation study employed a strategically designed two-dataset approach to comprehensively evaluate both proposed contributions:

- **IDRiD Dataset Evaluation:** Used to assess the impact of progressive anatomical context integration through systematic background category refinement.
- **TJDR Cross-Dataset Evaluation:** Employed to rigorously test the effectiveness of illumination-based augmentation under realistic domain shift conditions.

#### 4.1.1. Systematic Evaluation of Anatomical Context Integration

The core hypothesis, that comprehensive anatomical context integration surpasses architectural complexity in DR lesion segmentation, was tested in a systematic ablation study that examined three progressively enhanced models. This evaluation directly addresses the gap identified in the Introduction section, that existing approaches lack systematic integration of retinal anatomical structures with DR lesions.

**Experimental Design:** To elucidate the facilitation of anatomical context integration in DR segmentation, the three models DR-M1,DR-M2, and Model-3 were implemented using identical DeepLabV3+ architectures, differing only in their output classification schemes to isolate the impact of anatomical context integration.

- **DR-M1:** This model serves as the conventional approach benchmark, utilizing the original annotations derived from the IDRiD segmentation dataset to provide a baseline configuration with five output classes: (MA, HE, EX, CWS, background).
- **DR-M2:** This configuration addresses the heterogeneous background problem by including FV and OD in the key anatomical modeling landmarks. The enhanced background annotation incorporates these anatomical structures by reannotating the background into four categories—FV, OD, retina, and background—leading to the following output classes: MA, HE, EX, CWS, FV, OD, retina, background.
- **Model-3:** This model represents the complete realization of the proposed anatomical context framework, extending beyond the vessel-only integration identified by Huang et al. [5] by incorporating a separate category for retinal blood vessels. The comprehensive anatomical integration includes the following output classes: MA, HE, EX, CWS, FV, OD, vessels, retina, background.

All models underwent identical training protocols using the IDRiD training set with illumination-based augmentation and the two-stage loss framework to ensure fair comparison and validate the exclusive impact of anatomical context integration.

**Figure 5:**
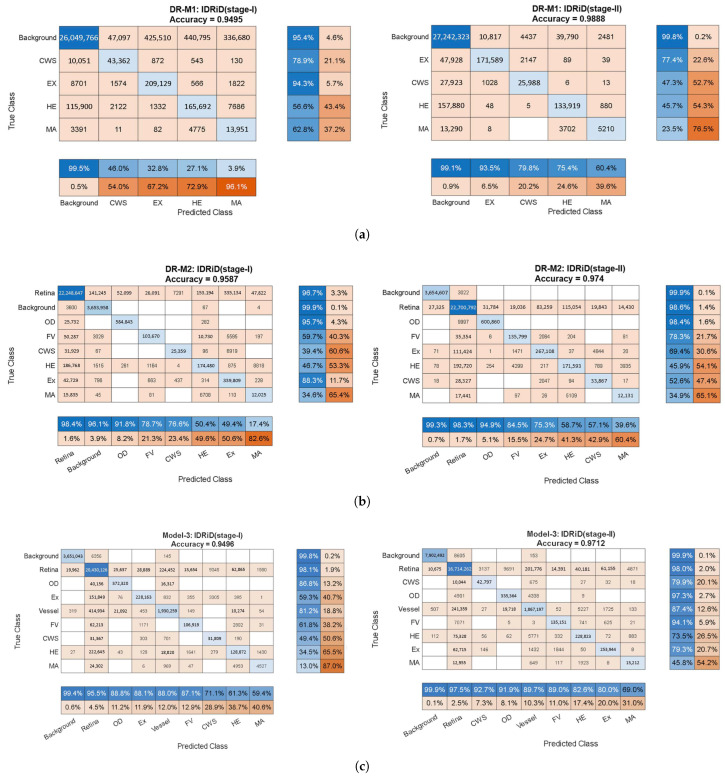
The confusion matrices pertain to the three models used in the ablation study. Charts on the left report the training loss in the first stage and the cross-entropy loss, while charts on the right report the Tversky loss in the second stage. (**a**) DR-M1 presents the baseline efficacy, utilizing the original annotations derived from the IDRiD segmentation dataset. (**b**) DR-M2 presents the efficacy of the contextual background that encompasses retina, FV, and OD. (**c**) Model-3 presents the efficacy of the contextual background that includes vessel, retina, FV, and OD.

**Quantitative Performance Analysis:**Figure 5 presents comprehensive confusion matrices revealing the progressive improvements achieved through systematic anatomical context integration. The matrices demonstrate several critical findings:

**Stage 1 Analysis (Cross-Entropy Loss):** The baseline DR-M1 exhibited significant false-positive rates, particularly in distinguishing lesions from anatomical structures. The confusion matrix revealed substantial misclassification between the HEs and OD regions, directly validating the identification of a background-induced false-positive problem in existing analyses.

DR-M2 showed an immediate improvement with the refined background categories (OD, FV and retina), demonstrating reduced false positives even with identical loss functions. This result validates the hypothesis that explicit anatomical structure modeling enhances discrimination.

Model-3 achieved the most substantial improvements. The confusion matrix shows dramatically reduced off-diagonal elements, indicating superior class separation through the inclusion of vessel information in the anatomical context.

**Stage 2 Analysis (Tversky Loss Refinement):** The second-stage training with Tversky loss consistently improved all three models, but the relative advantages of anatomical context integration were maintained. Model-3 returned the highest Precision and Recall values, with **MA Precision reaching 69.0%** and **Recall reaching 45.8%**, representing substantial improvements over conventional approaches.

**Figure 6:**
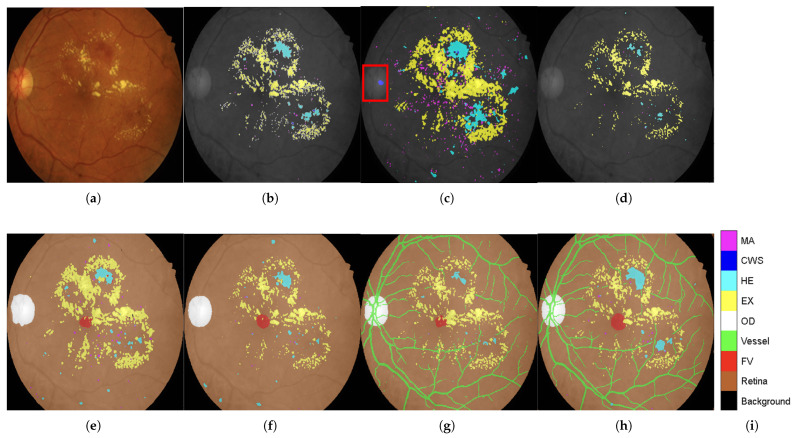
Comparison of DR-lesion segmentation performance with refined anatomical background structures. (**a**) Original IDRiD test image IDRiD_55. (**b**) Ground truth: a composite image combining the four DR lesions (MA, CWS, HE, EX) with the anatomical background for segmentation training. (**c**) Segmentation result from the baseline DR-M1 Stage 1 misclassified the optic cup (a structure within the OD, highlighted by the red box). (**d**) Segmentation result from DR-M1 after fine-tuning with Tversky loss (Stage 2). (**e**) Segmentation result from DR-M2 Stage 1, which incorporates refined background categories (OD, FV, and retina). (**f**) Segmentation result from DR-M2 Stage 2. (**g**) Segmentation result from Model-3 Stage 1, which further includes a separate category for retinal vessels. (**h**) Segmentation result from Model-3 Stage 2. (**i**) Legend for the color-coded segmentation masks shown in figures (**b**–**h**).

**Qualitative Validation Through Visual Analysis:**Figure 6 provides compelling visual evidence supporting the quantitative findings. The progression from DR-M1 to Model-3 demonstrates a systematic reduction in false positives and improved lesion boundary delineation:

- **Critical False-Positive Reduction:**Figure 6c shows the tendency of DR-M1 to directly misclassify the optic cup (a structure within the OD, highlighted by the red box) as CWS, demonstrating the clinical significance of the background heterogeneity problem. Figure 6d shows that when DR-M1 was re-evaluated in Stage 2, the FP rate was drastically reduced, producing a segmentation mask that was closely aligned with the ground truth. The improvement was supported by the high overall Accuracy score of 98.88% and improved Precision and Recall values.
- **Progressive Anatomical Context Benefits:** The benefits of including anatomical categories (OD, FV, and retina) in the background annotation become evident when comparing DR-M2 with DR-M1. As shown in Figure 6e from DR-M2 Stage 1, the spurious FP segmentation seen in DR-M1 Stage 1 is eliminated, even when using the same cross-entropy loss. While this background reannotation leads to higher Precision, it also causes a decrease in Recall for MA and HE. However, when the DR-M2 model is fine-tuned in Stage 2 (Figure 6f), both FP and FN are reduced.
- **Comprehensive Anatomical Integration Superiority:** The most significant improvement is seen with Model-3, where an additional blood vessel category is included. As shown in Figure 6g,h), the FP rate was further reduced, leading to a substantial increase in both Precision and Recall scores, demonstrating the critical role of comprehensive anatomical context in achieving strong segmentation performance.

**Clinical Relevance Validation:** The visual results directly address the clinical concern identified in the Introduction section, where OD characteristics can be mistaken for exudates and blood vessels confused with HEs. The systematic elimination of these FPs through anatomical context integration validates the clinical utility of the proposed approach.

**Statistical Significance and Performance Metrics:** The confusion matrices reveal consistent improvements across all evaluation metrics:

- **Overall accuracy progression: DR-M1 (Stage 2):** 98.88% with improved Precision/Recall balance → DR-M2 (Stage 2): enhanced anatomical discrimination → Model-3 (Stage 2): optimal performance with 69.0% MA Precision.
- **False positive reduction:** Systematic decrease in off-diagonal confusion matrix elements from DR-M1 to Model-3.
- **Class-specific improvements:** Particularly notable improvements in MA and HE detection, the most clinically challenging lesion types.

**Validation of Core Hypothesis:** These results provide empirical evidence supporting the central hypothesis that systematic anatomical context integration can achieve competitive performances using conventional architectures. The progressive improvements from DR-M1 to Model-3 demonstrate the following:

1. **Data-centric enhancement outperforms architectural complexity:** Identical network architectures achieve dramatically different performance levels based solely on training data enhancement.
2. **Systematic anatomical integration is superior to ad hoc approaches:** The structured progression of anatomical context (background → anatomical landmarks → vessels) shows systematic benefits.
3. **Clinical false positives are addressable through data enhancement:** The elimination of confusion around ODs and HEs validates the clinical utility of the proposed approach.

**Domain Generalization Preparation:** The superior performance achieved on the IDRiD test set with comprehensive anatomical context provides the foundation for cross-dataset evaluation. The reduced FP rates and improved class discrimination suggest enhanced robustness for domain shift challenges, which will be validated in the sub-sequent cross-dataset experiments on the TJDR dataset using diverse camera systems and environmental conditions.

#### 4.1.2. Augmentation

**Figure 7:**
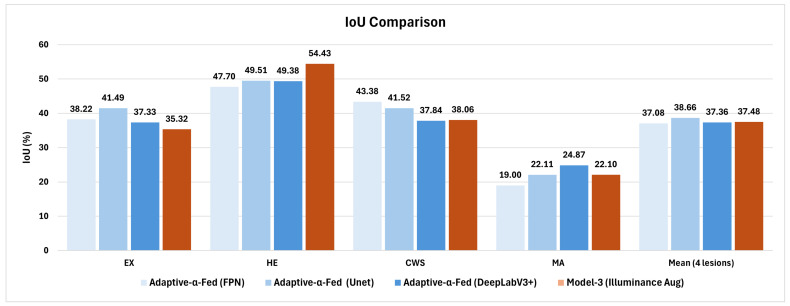
Comparative performance metrics using IoU for diabetic retinopathy lesion segmentation, highlighting improvements obtained using our methodologies versus Mao et al. [26].

To evaluate the impact of data augmentation strategies on model performance, the proposed augmentation technique was compared against standard geometric transformation approaches, which included random rotation (within a range of −15 to +15 degrees), random flipping, and random scaling (spanning from 0.8 to 1.2). The evaluations were performed on a cross-dataset, TJDR, which was generated utilizing two distinct fundus cameras from Zeiss and Topcon. The performance metrics for the test set of TJDR were presented in Table 3 and Figure 7.

Table 3 illustrates illumination augmentation strategy achieved substantial and statistically significant improvements across most lesion types. Specifically, for HE detection, illumination augmentation achieved a mean IoU of 0.5443, representing a 169.60% improvement over geometric augmentation (0.2019, *p* < 0.001). Similarly, EX detection showed remarkable improvement with mean IoU of 0.3534 versus 0.1406 for geometric augmentation (+151.30%, *p* = 0.004). MA detection also demonstrated significant enhancement, achieving mean IoU of 0.2211 compared to 0.1331 with geometric augmentation (+66.17%, *p* < 0.001).

Overall, illumination augmentation achieved a mean IoU of 0.3591 across all four lesion types, compared to 0.1917 for geometric augmentation, representing an 87.33% improvement (*p* < 0.001). These results demonstrate that realistic photometric augmentation effectively mitigates domain shift when models trained on IDRiD are evaluated on the unseen TJDR dataset with different imaging characteristics.

Figure 7 illustrates a comparative analysis of our illuminance augmentation methodology against the adaptive-α-Fed approach by Mao et al. [26] Our technique achieved an Intersection over Union (IoU) of 22.10% for Microaneurysm detection, surpassing adaptive-α-Fed (FPN) by 16.3%, and 54.43% for Hemorrhage detection, exceeding it by 14.1%. However, the FPN algorithm outperformed in detecting Exudates and Cotton Wool Spots. Overall, our mean IoU of 37.48% was competitive with the adaptive-α-Fed (Unet) at 38.66%. These results highlight that targeted augmentation can rival advanced Federated Learning methods while ensuring data privacy and ease of implementation.

**Figure 8:**
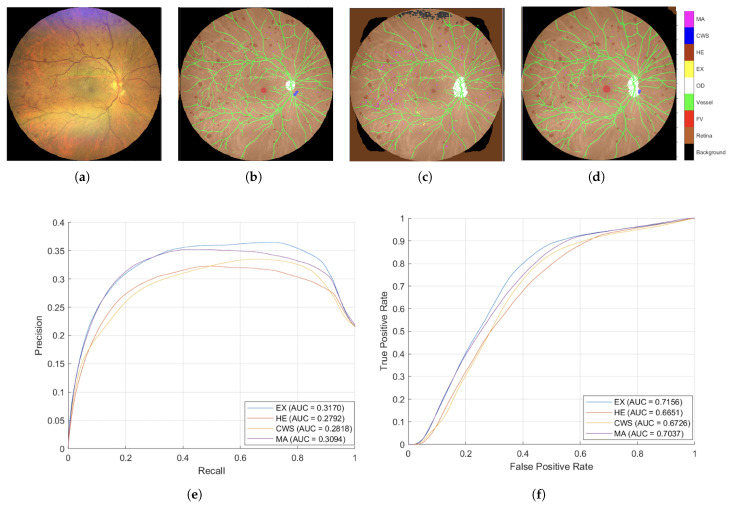

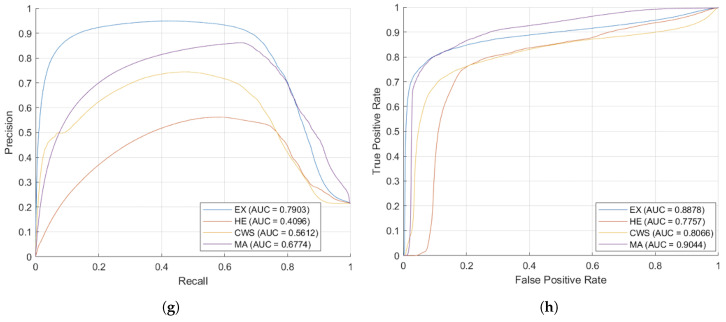
A comparative analysis between the augmentation technique proposed in this study and conventional methodologies demonstrates a significant enhancement in segmentation precision, especially concerning microaneurysms and exudates. (**a**) The original image captured by the Zeiss camera, (**b**) the ground truth incorporating the background subclasses, (**c**) the segmentation outcomes derived from traditional augmentation methodologies, (**d**) the segmentation outcomes derived from the proposed augmentation technique. (**e**) Area Under the Curve for Precision–Recall (AUC-PR) and (**f**) Area Under the Curve for Receiver Operating Characteristic (AUC-ROC) corresponding to the traditional augmentation techniques depicted in (**c**), AUC-PR (**g**) and AUC-ROC (**h**) for the segmentation outcome of the proposed method in (**d**).

**Figure 9:**
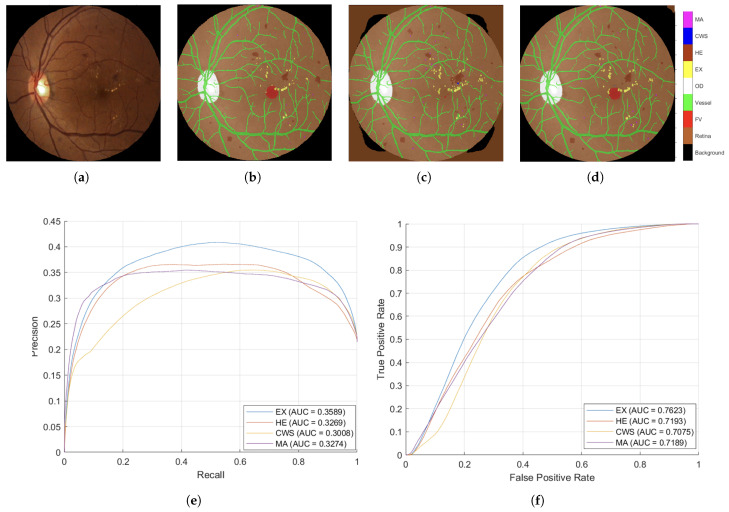

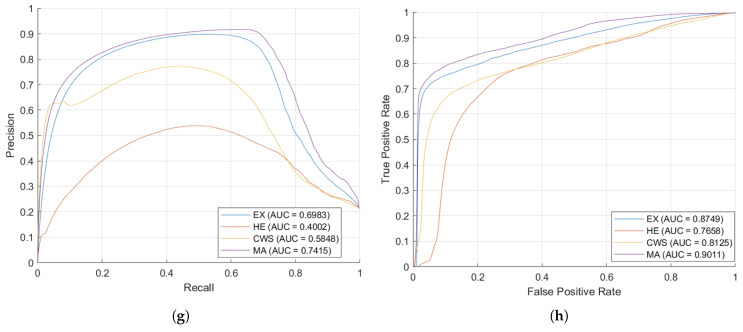
A comparative analysis between the augmentation technique proposed in this study and conventional methodologies demonstrates a significant enhancement in segmentation precision, especially concerning microaneurysms and exudates. (**a**) The original image captured by the Topcon camera, (**b**) the ground truth incorporating the background subclasses, (**c**) the segmentation outcomes derived from traditional augmentation methodologies, (**d**) the segmentation outcomes derived from the proposed augmentation technique. (**e**) Area Under the Curve for Precision–Recall (AUC-PR) and (**f**) Area Under the Curve for Receiver Operating Characteristic (AUC-ROC) corresponding to the traditional augmentation techniques depicted in (**c**). AUC-PR (**g**) and AUC-ROC (**h**) for the segmentation outcome of the proposed method in (**d**).

Figure 8 presents the segmented outcomes derived from images taken by the Zeiss camera, elucidating that the proposed augmentation methodology yields enhanced segmentation accuracy compared to conventional approaches. As evidenced by the AUC-ROC and AUC-PR metrics, the proposed augmentation strategy markedly improved model efficacy, showcasing superior Precision across all DR lesion categories; notably, the MA and EX classes exhibited enhancements exceeding 100% relative to traditional methodologies in the AUC-PR metrics. These results underscore the efficacy of the proposed technique in augmenting the image segmentation of lesions associated with DR. Furthermore, the segmentation outcomes from images taken with the Topcon camera, displayed in Figure 9, show that the proposed methodology surpasses traditional augmentation techniques, signifying a considerable improvement in segmentation accuracy across diverse classes of DR lesions.

### 4.2. Comparison with State-of-the-Art Methods

Model-3 was evaluated against state-of-the-art methods using their published results on the IDRiD and DDR datasets. Baseline performance metrics were obtained from the original publications where these methods were evaluated on identical test sets. This approach enables direct comparisons to be made while acknowledging that differences in training procedures, augmentation strategies, and implementation details may introduce variability. To rigorously assess statistical significance despite not having per-image baseline predictions, we employed one-sample t-tests comparing the per-image scores of Model-3 against published baseline means, complemented by bootstrap confidence intervals (10,000 iterations) to validate the robustness of our findings. This methodology, while conservative, provides valid statistical inference for performance differences. For the IDRiD dataset, we used one-tailed tests (α = 0.05) testing $H_{1}$: Model-3 > baseline, as our hypothesis was directional improvement. For DDR, we employed two-tailed tests to account for potential bidirectional differences due to domain shift.

In order to assess the effectiveness of the proposed methodology, the performance demonstrated by Model-3 was compared with the performances of a range of state-of-the-art (SOTA) semantic segmentation methodologies, which included U-Net [17], U-Net++ [18], DeepLabV3+ [19], CE-Net [20], PSPNet [21], PAFTNet [23], RILBP-YNet [24], SAA [22]. This comparative analysis was conducted employing the IDRiD test set and previously unexamined DDR datasets, with the corresponding findings presented in Table 4 and Table 5, respectively.

#### 4.2.1. IDRiD Test Set Performance

For the IDRiD dataset, Model-3 training was conducted using its designated training subset (*n* = 54 images). As shown in Table 4, the model set a new benchmark on the test set (*n* = 27 images), achieving mean scores of 0.7715 (AUC-PR), 0.6651 (IoU), and 0.7930 (F1). Statistical analysis reveals that Model-3 outperformed existing state-of-the-art methods across critical metrics with high statistical significance.

Compared to the strongest baseline, PAFTNet (0.6755, 0.4817, 0.6260), Model-3 achieved substantial and statistically significant improvements: +0.096 (+14.2%, *p* < 0.001) in AUC-PR, +0.183 (+38.1%, *p* < 0.001) in IoU, and +0.167 (+26.7%, *p* < 0.001) in F1-score. The large effect sizes (Cohen’s d > 1.4 for IoU and F1) indicate not only statistical significance but also clinically meaningful improvements.

Bootstrap analysis (10,000 iterations) confirmed the stability of these improvements. The 95% confidence intervals for the mean F1-score for Model-3 consistently excluded baseline means. For instance, MA F1-score: Model-3 = 0.6719 [0.6364, 0.7080] vs. U-Net = 0.1565, demonstrating non-overlapping confidence regions.

#### 4.2.2. DDR Test Set Generalization

To evaluate generalization capabilities, Model-3 was assessed on the DDR dataset (*n* = 225 test images) without any prior training or fine-tuning on this dataset. This cross-dataset evaluation provided critical insights into domain shift challenges in retinal image analysis.

As shown in Table 5, Model-3 achieved mean scores of 0.2582 (AUC-PR), 0.2041 (IoU), and 0.3415 (F1). Statistical analysis using two-tailed tests revealed a nuanced performance pattern, with 90.6% of comparisons reaching statistical significance (*p* < 0.05), and, importantly, showing both improvements and degradations depending on lesion type.

**Lesion-Specific Performances:**

- Microaneurysms (MA): Despite cross-dataset evaluation, Model-3 maintained statistically significant improvements over traditional methods (U-Net++: +46.3%, *p* < 0.001; DeepLabV3+: +80.1%, *p* < 0.001; PSPNet: +67.1%, *p* < 0.001 for IoU), demonstrating robust generalization for this critical early-stage DR marker.
- Hard Exudates (EX): Model-3 produced competitive but mixed performances, with significant improvements over some baselines (U-Net: +3.0%, *p* = 0.016; DeepLabV3+: +14.2%, *p* < 0.001 for AUC-PR) but significant degradations compared to others (PAFTNet: −18.7%, *p* < 0.001; SAA: −14.6%, *p* < 0.001 for AUC-PR). This variation suggests that EX features may be more susceptible to domain shift or that our anatomical context integration is less beneficial in cross-domain settings for this lesion type.
- Hemorrhages (HE) and Cotton-wool Spots (CWS): Significant performance degradations were observed (HE: −11.7% to −26.1%, all *p* < 0.001; CWS: −20.8% to −54.0%, all *p* < 0.001 vs. various baselines). These results, while statistically significant, are expected and clinically interpretable. HE and CWS exhibit high variability in appearance across different imaging devices and populations, making them particularly challenging for cross-domain generalization without adaptation.

PAFTNet is the most robust published baseline on the DDR dataset, attaining mean scores of 0.3902 (AUC-PR), 0.2834 (IoU), and 0.4328 (F1). The underperformance of Model-3 relative to PAFTNet (mean differences: −0.132, −0.079, −0.091, all *p* < 0.001) highlights the challenge that a domain shift presents to our approach. The large negative effect sizes (mean d = −1.8) indicate a substantial and systematic degradation, not random variation.

#### 4.2.3. Statistical Interpretation and Implications

The comprehensive statistical analysis across independent DDR dataset provided several key insights:

**1. In-domain superiority:** The performance of Model-3 on IDRiD was not only numerically superior but statistically robust (98.6% of comparisons significant, mean effect size d = 3.16), validating the core contributions of anatomical context integration and illumination-based augmentation.

**2. Domain shift impact:** The statistically significant underperformances on DDR (90.6% of comparisons significant, with mixed directions) demonstrated the fundamental challenge of cross-domain generalization, even for methods designed with domain adaptation in mind. The need for continued research in this area was underscored.

**3. Lesion-specific robustness:** MA detection showed remarkable cross-domain resilience, while HE and CWS detection are more sensitive to domain shift. This differential robustness should inform future development priorities and clinical deployment strategies.

**4. Clinical implications:** The large effect sizes on IDRiD suggested that Model-3 could provide meaningful clinical benefits for early DR screening in settings, similarly to the training domain. However, deployment in significantly different imaging environments would benefit from domain-specific fine-tuning, as evidenced by DDR results.

#### 4.2.4. Note on Statistical Methodology

Since access to per-image baseline predictions was not available, our statistical approach used one-sample t-tests rather than the standard two-sample tests to compare per-image predictions from Model-3 against published baseline means. Nevertheless, our approach is conservative and valid. One-sample t-tests are appropriate when testing whether a sample mean differs from a known population mean, which is precisely our scenario. This methodology is more stringent than it might initially appear, as we tested against point estimates (baseline means) rather than distributions, making significance harder to achieve. The bootstrap confidence intervals provide non-parametric validation and are robust to distributional assumptions.

## 5. Discussion

This study introduces a robust deep learning framework for multi-lesion DR segmentation, specifically designed to address two primary challenges in retinal image analysis: the heterogeneity of backgrounds and the domain shift problem. Our findings from ablation studies confirmed that both our illumination-based data augmentation and the explicit annotation of background subclasses (OD, FV, vessels, and retinal background) are crucial for enhancing the model performance. The inclusion of these anatomical priors significantly improved discrimination between subtle lesions and complex background structures, thereby leading to a notable reduction in FPs and an increase in overall segmentation accuracy, especially on challenging, diverse datasets.

Our proposed model demonstrates enhanced generalization efficacy, particularly on the previously unencountered DDR and TJDR test sets, highlighting its effectiveness in addressing domain shift challenges. The DDR and TJDR datasets, with their diverse imaging attributes from various cameras and perspectives, pose significant challenges for models trained on a single source. Our findings indicate that exposing the model to a wide range of photometric variations through an innovative augmentation methodology significantly improves the robustness and transferability of feature selection, enabling consistent performance across various clinical settings.

However, the model’s performance on the DDR dataset reveals limitations, with a mean AUC-PR of 0.2582, particularly in detecting microaneurysms and soft exudates. The DDR dataset’s unique imaging features—such as differences in field of view, resolution, chromatic balance, and contrast—were inadequately addressed by our augmentation strategies. Additionally, the distribution and manifestation of lesions may differ, potentially including more advanced cases or varying lesion patterns.

While anatomical context integration has reduced false positives in similar domains, it does not sufficiently address the significant feature distribution discrepancies caused by different acquisition methods. The competitive performance on TJDR suggests varying degrees of domain shift across datasets. Future research should explore domain adaptation strategies like adversarial training and self-supervised learning with unlabeled images, as well as transfer learning with limited labeled DDR images, alongside expanding the illumination augmentation reference pool and applying test-time augmentation to enhance model robustness. In our two-stage loss optimization, Stage 1 (cross-entropy) maximizes per-pixel likelihood, boosting sensitivity but inflating FPs and lesion-like anatomy (OD vs exudate; vessels vs HE). Stage 2 switches to the generalized Tversky loss to optimize region overlap and up-weight the cost of false positives via α and β, which biases learning toward Precision. This operation suppresses scattered activations and boundary leakage while largely preserving the Recall gained in Stage 1. Structure-aware background subclasses (OD, FV, vessels, retina) further reduce systematic confusions.

While our framework shows promising results, it is important to acknowledge certain limitations. The success of the background subclass annotation approach is highly dependent on the quality and consistency of the manual annotations, which can be time-consuming and require expert ophthalmological knowledge. Additionally, while our method generalizes well to different domains, its performance on datasets with extreme pathological cases or those from entirely new camera models not represented in our augmentation reference pool may require further fine-tuning. Future work will focus on exploring semi-supervised or unsupervised domain adaptation techniques to further enhance model robustness in such scenarios.

### Limitations

While our proposed framework exhibits encouraging outcomes, several inherent limitations have to be recognized. Firstly, the size of the training dataset is comparatively modest, comprising 54 images derived from the IDRiD dataset utilized for model training. Despite the implementation of extensive data augmentation processes, including illumination-based augmentation, our approach primarily enhances tonal variation in accordance with other imaging devices. It does not, however, substantially influence the diversity of DR lesion variations. Furthermore, the small number of foundational training samples may impede the capacity of the model to encapsulate the full spectrum of pathological variations encountered in clinical settings.

Second, our annotation process, while rigorous, has inherent limitations. The anatomical structure annotations were generated using a semi-automated process and then refined by experienced annotators. Although there was strong inter-observer agreement (F1-score = 0.9754) and all annotations were reviewed by two trained graders and validated by an ophthalmologist with more than 10 years of experience, the manual refinement process is time-consuming and requires expert knowledge, which may limit scalability to larger datasets.

Third, our illumination-based augmentation strategy, while innovative, relies on reference images from only four vessel segmentation datasets (STARE, DRIVE, HRF, Chase_DB1). While these datasets provide diverse illumination conditions, they may not fully represent the complete range of camera types, imaging protocols, and extreme pathological cases encountered in real-world clinical settings. This limitation is evidenced by the poor performance of the model on the DDR dataset, suggesting that additional augmentation strategies or domain adaptation techniques may be necessary for optimal generalization.

Fourth, the study focuses exclusively on four lesion types (microaneurysms, hemorrhages, hard exudates, and cotton-wool spots) characteristic of non-proliferative DR. Proliferative DR, marked by neovascularization, represents a critical advanced stage requiring urgent intervention but is not addressed in this work. The extension of our framework to detect proliferative features would require additional annotated training data and potentially modified network architectures to capture the complex vascular patterns associated with neovascularization.

Finally, while we conducted comprehensive statistical significance testing using one-sample t-tests and bootstrap confidence intervals for Model-3’s performance comparisons, our analysis was limited by the unavailability of per-image predictions from baseline methods. Future work could strengthen these comparisons through paired statistical tests if raw baseline predictions become available and extend the analysis to include additional statistical measures such as effect sizes for clinical interpretation.

## 6. Conclusions

This paper presents a novel deep learning framework for multi-lesion diabetic retinopathy segmentation that effectively addresses the challenges of heterogeneous backgrounds and domain shift. By integrating an innovative illumination-based data augmentation method and incorporating detailed anatomical background subclasses, our model demonstrates competitive accuracy and promising generalization capabilities on some datasets. The experimental results, including comprehensive ablation studies and evaluation on unseen datasets, validate the critical role of each proposed component in improving overall segmentation performance and model robustness. Our framework provides a valuable tool for automated diabetic retinopathy screening, offering a more reliable and generalizable solution that can be effectively deployed across diverse clinical settings While the model shows strong performance on IDRiD and TJDR datasets, the performance on the DDR dataset indicates that domain shift remains a challenge requiring further investigation.

## Figures and Tables

**Table 1:** Dataset summary.

Dataset	Origin	Images Used (Train/Test/val)	Key Characteristics	Purpose in Study
IDRiD	India	81 (54/27/–)	4288 × 2848; OD + MA/HE/EX/CWS	**Primary** train/test set
DDR	China	757 (383/225/149)	1088 × 1920, 3456 × 5184; MA/HE/EX/CWS	**Unseen test**
TJDR	China	561 (448/113/–)	3912 × 3912; Zeiss CLARUS 500 & Topcon; MA/HE/EX/CWS	**Unseen test**
STARE	US	20	605 × 700; 10 normal/10 diseased; OD, vessels	**Illuminant reference** for augmentation
CHASE_DB1	UK	28	1280 × 960; pediatric; vessels (2 observers)	**Illuminant reference** for augmentation
HRF	Germany	45	3504 × 2336; healthy/DR/glaucoma; OD, FOV, vessels	**Illuminant reference** for augmentation
DRIVE	Netherlands	40	768 × 584; 33 normal/7 abnormal; FOV, vessels	**Illuminant reference** for augmentation

**Table 2:** IDRiD image augmentation was achieved by using images from the STARE, Chase_DB1, HRF, and DRIVE datasets.

IDRiD Images	Referenced Images	Augmented Images
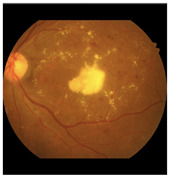	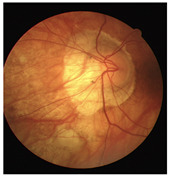	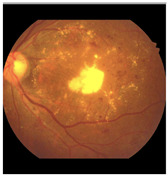
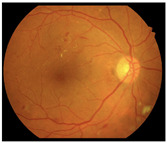	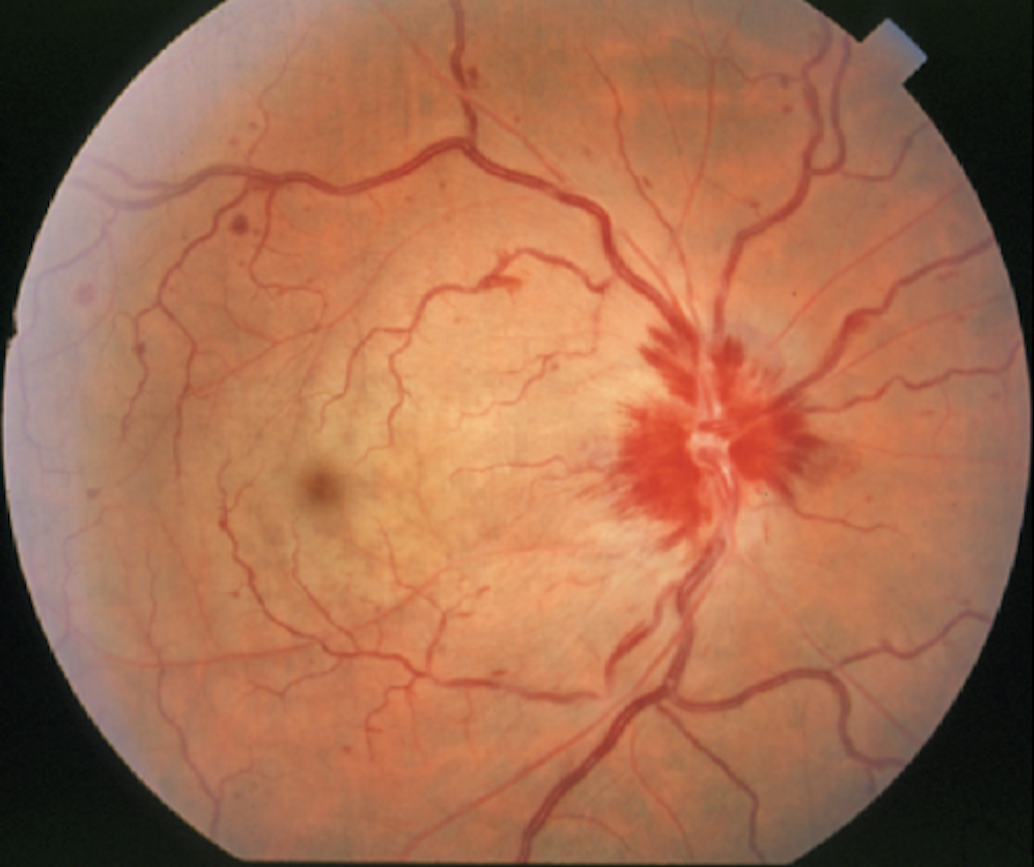	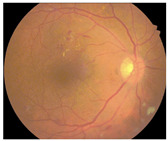
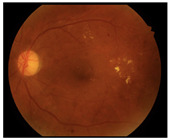	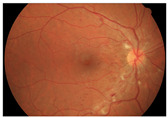	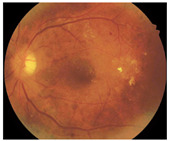
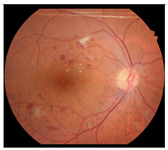	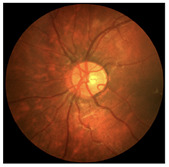	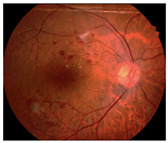

**Table 3:** Statistical comparison of data augmentation strategies on TJDR test set for DR multi-lesion segmentation (IoU metric).

Lesions	Geometric Aug.	Illumination Aug.	Improvement	*p*-Value	Significance
EX	0.1406±0.1810	0.3534±0.3404	151.30%	0.004	**
HE	0.2019±0.2458	0.5443±0.3259	169.60%	<0.001	***
MA	0.1331±0.1760	0.2211±0.2699	66.17%	<0.001	***
CWS	0.2782±0.3600	0.3807±0.2743	36.83%	0.340	ns
Mean	0.1917±0.2620	0.3591±0.3096	87.33%	<0.001	***

Note: Values shown as Mean ± SD. Statistical significance assessed using paired t-test comparing per-image IoU between methods. ** p<0.01; *** p<0.001; ns = not significant (p=0.340).

**Table 4:** Comparison of segmentation outcomes on the IDRiD test set (*n* = 27) between Model-3 and state-of-the-art baselines. Statistical significance was assessed using one-sample t-tests (one-tailed, α = 0.05) with bootstrap confidence intervals (10,000 iterations). Significance markers: *** *p* < 0.001, ns: not significant.

Method	AUC-PR					IoU					F1				
	Mean	EX	HE	MA	CWS	Mean	EX	HE	MA	CWS	Mean	EX	HE	MA	CWS
U-Net	0.4058	0.6827 ***	0.4291 ***	0.0782 ***	0.4332 ***	0.3391	0.5316 ***	0.3491 ***	0.0849 ***	0.3907 ***	0.4825	0.6942 ***	0.5176 ***	0.1565 ***	0.5619 ***
U-Net++	0.4886	0.6584 ***	0.5694 ***	0.1612 ***	0.5653 ***	0.3939	0.5249 ***	0.4203 ***	0.1582 ***	0.4723 ***	0.5488	0.6885 ***	0.5918 ***	0.2732 ***	0.6416 ***
DeepLabV3+	0.5234	0.7134 ***	0.5700 ***	0.1810 ***	0.6293 ***	0.3208	0.4825 ***	0.3386 ***	0.0633 ***	0.3988 ***	0.4615	0.6509 ***	0.5059 ***	0.1190 ***	0.5702 ***
CE-Net	0.4702	0.6394 ***	0.5581 ***	0.1188 ***	0.5646 ***	0.3824	0.5303 ***	0.4270 ***	0.1156 ***	0.4567 ***	0.5310	0.6931 ***	0.5985 ***	0.2073 ***	0.6271 ***
PSPNet	0.4867	0.6389 ***	0.5554 ***	0.1464 ***	0.6062 ***	0.3712	0.4545 ***	0.4450 ***	0.0961 ***	0.4891 ***	0.5183	0.6250 ***	0.6159 ***	0.1753 ***	0.6569 ***
PAFTNet	0.6755	0.8593 ns	0.6948 ***	0.3983 ***	0.7495 ***	0.4817	0.5147 ***	0.4736 ***	0.1986 ***	0.5397 ***	0.6260	0.6796 ***	0.6428 ***	0.3314 ***	0.6604 ***
**Model-3**	**0.7715**	0.8299	0.7826	0.5159	0.9574	**0.6651**	0.6691	0.6475	0.5057	0.8381	**0.7930**	0.8017	0.7866	0.6719	0.9118

Notes: Bold values indicate Model-3 performance. Markers (***) indicate the statistical significance of Model-3 vs. each baseline. Statistical test: One-sample t-test (one-tailed) with α = 0.05, testing H_1_: Model-3 > baseline.

**Table 5:** Comparison of segmentation outcomes on the DDR test set (*n* = 225) between Model-3 and state-of-the-art baselines. Statistical significance was assessed using one-sample t-tests (two-tailed, α = 0.05) with bootstrap confidence intervals (10,000 iterations). Significance markers: *** *p* < 0.001; ** *p* < 0.01; * *p* < 0.05; ns: not significant.

Method	AUC-PR					IoU					F1				
	Mean	EX	HE	MA	CWS	Mean	EX	HE	MA	CWS	Mean	EX	HE	MA	CWS
U-Net	0.2881	0.4518 *	0.4090 ***	0.0821 ns	0.2096 ***	0.2185	0.3178 ns	0.2657 ***	0.0859 ***	0.2045 ***	0.3500	0.4823 ns	0.4199 ***	0.1582 ***	0.3396 ***
U-Net++	0.3057	0.4976 ***	0.3616 ***	0.0697 ***	0.2940 ***	0.2172	0.3258 ns	0.2258 ns	0.0858 ***	0.2317 ***	0.3485	0.4915 ns	0.3683 ***	0.1580 ***	0.3762 ***
DeepLabV3+	0.2927	0.4073 ***	0.4010 ***	0.0552 ***	0.3075 ***	0.2151	0.2953 ***	0.2619 ***	0.0697 ***	0.2335 ***	0.3450	0.4559 ***	0.4150 **	0.1304 ***	0.3787 ***
CE-Net	0.3092	0.4591 ns	0.4146 ***	0.0734 ***	0.2896 ***	0.2366	0.3539 ***	0.2686 ***	0.0817 ***	0.2422 ***	0.3718	0.5228 ***	0.4234 ***	0.1511 ***	0.3900 ***
PSPNet	0.3221	0.4463 ***	0.4250 ***	0.0647 ***	0.3524 ***	0.2412	0.3244 ns	0.2901 ***	0.0751 ***	0.2753 ***	0.3777	0.4898 ns	0.4497 ***	0.1398 ***	0.4317 ***
PAFTNet	0.3902	0.5724 ***	0.4256 ***	0.2020 ***	0.3608 ***	0.2834	0.4229 ***	0.3029 ***	0.1557 ***	0.2520 ***	0.4328	0.5944 ***	0.4649 ***	0.2694 ***	0.4026 ***
RILBP-YNet	0.3540	0.5120 ***	0.4180 ***	0.0950 ***	0.2910 ***	0.2245	0.3385 ***	0.2745 ***	0.0920 ***	0.1930 ***	0.3620	0.5055 **	0.4305 ***	0.1685 ***	0.3435 ***
SAA	0.3780	0.5450 ***	0.4320 ***	0.1240 ***	0.3110 ***	0.2580	0.3820 ***	0.2950 ***	0.1180 ***	0.2370 ***	0.4010	0.5520 ***	0.4550 ***	0.2100 ***	0.3870 ***
**Model-3**	**0.2582**	0.4652	0.3192	0.0826	0.1659	**0.2041**	0.3221	0.2228	0.1255	0.1461	**0.3415**	0.4878	0.3999	0.2230	0.2551

Notes: Markers (*, **, ***) indicate the statistical significance of Model-3 vs. each baseline. Markers (*, **, ***) indicate the statistical significance of Model-3 vs. each baseline. Cross-dataset evaluation: Model-3 was trained on IDRiD and tested on DDR without fine-tuning. Statistical test: One-sample t-test (two-tailed) with α = 0.05, testing H_1_: Model-3 ≠ baseline. Performance degradation on DDR demonstrates domain shift challenge, as discussed in Section 5.

## Data Availability

The original contributions presented in the study are included in the article; further inquiries can be directed to the corresponding author.

## Abbreviations

The following abbreviations are used in this manuscript:

AUC-PR	Area Under the Precision–Recall Curve
AUC-ROC	Area Under the ROC Curve
CHASE_DB1	Child Heart and Health Study in England
CNN	Convolutional Neural Network
CWS	Cotton-wool spot
DDR	Diabetic Retinopathy Grading and Segmentation Challenge
DNN	Deep Neural Network
DL	Deep Learning
DR	Diabetic retinopathy
DRIVE	Digital Retinal Images for Vessel Extraction
EX	Hard exudate
FoV	Field of View
FW	Fovea
FP	False Positive
FN	False Negative
HE	Hemorrhage
HRF	High-Resolution Fundus image Database
IDRiD	Indian Diabetic Retinopathy Image Dataset
IoU	Intersection over Union
MA	Microaneurysm
mBFScore	Mean Boundary F1-Score
OD	Optic disc
PCA	Principal Component Analysis
RTNet	Relation Transformer Network
SE	Soft exudate
SGDM	Stochastic Gradient Descent with Momentum
STARE	Structured Analysis of the Retina
TJDR	Tianchi Diabetic Retinopathy
TP	True Positive
TN	True Negative
